# Supplementary material for: Thinking inside the box: Restoring the propolis envelope facilitates honey bee social immunity
Source: PLoS One. 2024 Jan 31;19(1):e0291744. doi: 10.1371/journal.pone.0291744 (PMC10830010; doi:10.1371/journal.pone.0291744)
Supplement: S1 Fig — Four volunteers were provided reference photos (A) demonstrating what propolis looks like and differentiating wax from propolis. Volunteers were instructed to score photos on a scale from 1–10, based on % coverage of propolis, not on background coloration of the box or comb where it attached (B). Volunteers then used a Google form to fill out a practice survey, which allowed them to view and score ten sample photos. Finally, volunteers completed a full survey, scoring each wall of each box (C). Scores from all four walls, and from all four volunteers were averaged to create to create a composite “propolis score” for each colony. (PDF) [file pone.0291744.s001.pdf]

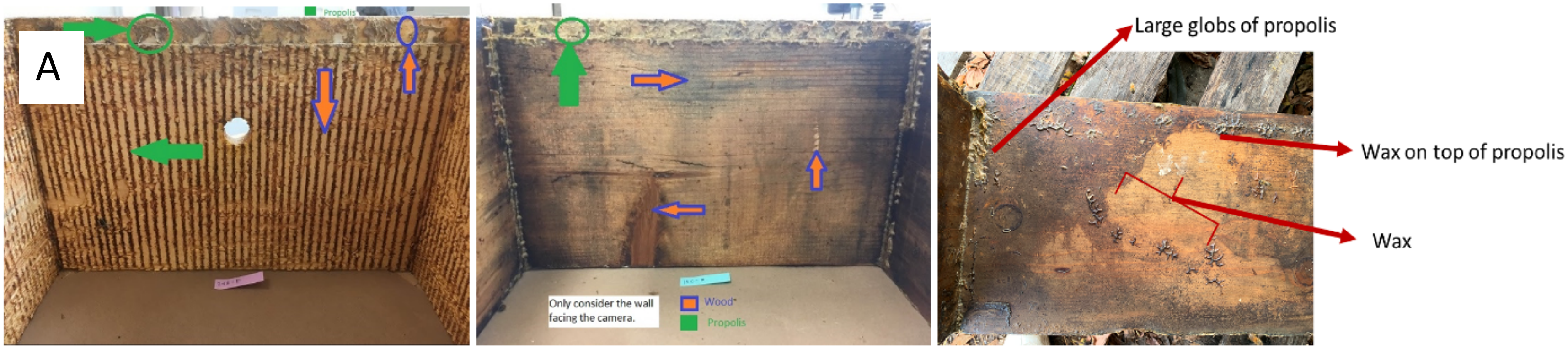

B

Propolis experiment real deal survey

Thank you for completing the practice survey. Please fill out this real deal survey to help us understand how much propolis honey bees need in their nest environment in order to experience the health benefits that this medicinal material provides.

On a scale from one to ten, visually rate the amount of propolis found in the walls featured in the following photos. Note that each photo features one wall. Other walls will be visible, but just base your estimate on the featured wall.

There are a couple of photos of black propolis traps that still contained propolis after we removed them from the walls. Please score these the same way you would score a full wall.

Some boxes are discolored; sometimes propolis is difficult to distinguish from wax. Don't worry too much; just do your best! Try not to spend more than 10 seconds per photo.

Thank you for helping us learn about the benefits of propolis to honey bee health!

C

Propolis deposition scoring guide

1- no propolis – 10% coverage

2- 10-20% propolis coverage

3- 20-30% propolis coverage

4- 30-40%

5- 40-50%

6- 50-60%

7- 60-70%

8- 70-80%

9- 80-90% propolis coverage

10- 90-100% propolis coverage

Figure S1. Propolis scoring methods. Four volunteers were provided reference photos (A) explaining what propolis looks like and differentiating wax from propolis. Volunteers were instructed to score photos on a scale from 1-10, based on % coverage of propolis, not on background coloration of the box or comb where it attached (B). Volunteers then used a Google form to fill out a practice survey, which allowed them to view and score ten sample photos. Finally, volunteers completed a full survey, scoring each wall of each box (C). Scores from all four walls, and from all four volunteers were averaged to create to create a composite "propolis score" for each colony.
