# Supplementary material for: Thinking inside the box: Restoring the propolis envelope facilitates honey bee social immunity
Source: PLoS One. 2024 Jan 31;19(1):e0291744. doi: 10.1371/journal.pone.0291744 (PMC10830010; doi:10.1371/journal.pone.0291744)
Supplement: S2 Fig — Control, trap, and rough box hive bodies were evaluated by four volunteers. The black text box at the upper left corner of each photo indicates the score assigned to that photo, according to one volunteer. (PDF) [file pone.0291744.s002.pdf]

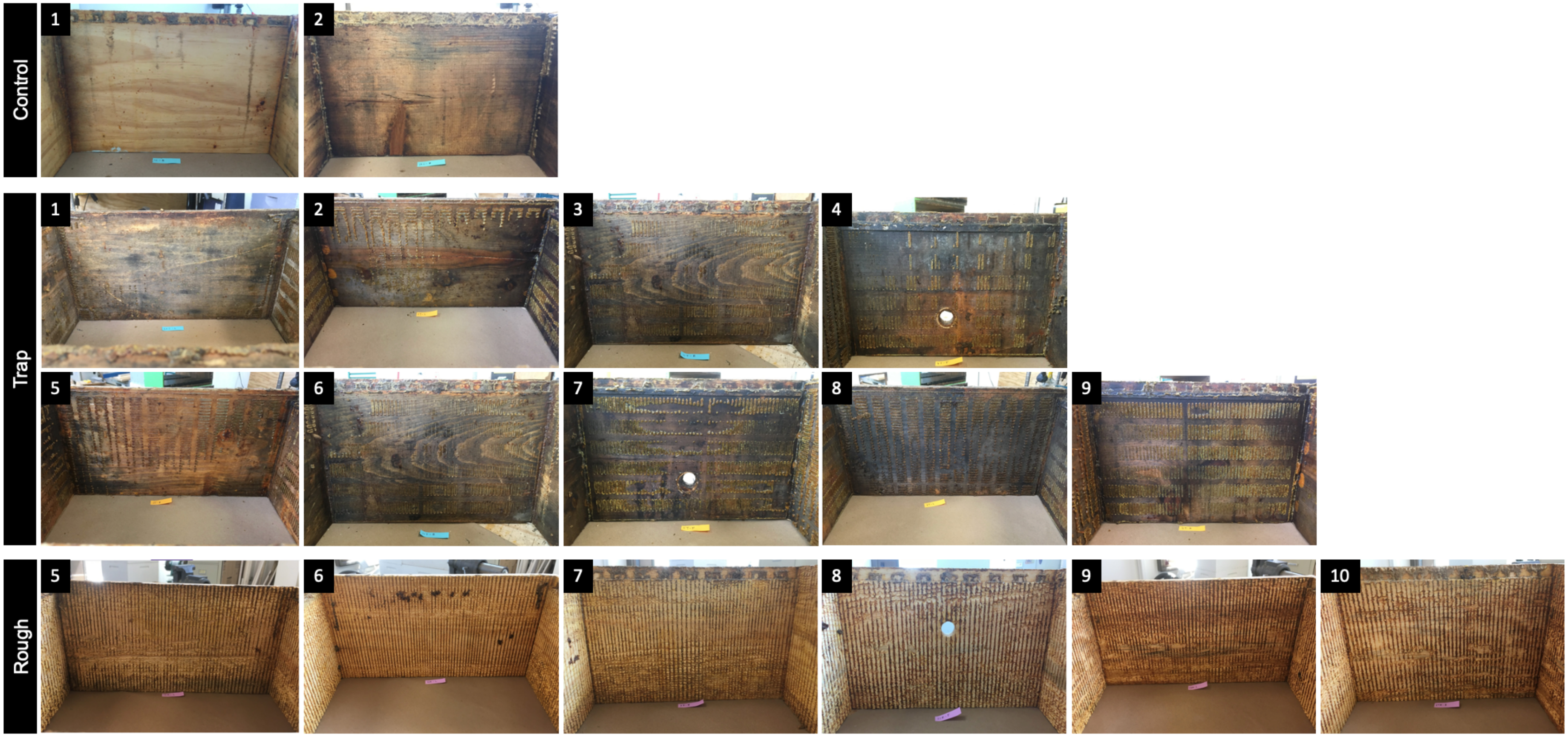

Figure S2. Sample of propolis scoring results. Control, trap, and rough box hive bodies were evaluated by four volunteers. The black text box at the upper left corner of each photo indicates the score assigned to that photo, according to one volunteer.
